# The Fifteenth International Congress of Medicine, Lisbon, 1906

**Published:** 1906-03-17

**Authors:** 


### HOSPITAL ADMINISTRATION.

### CONSTRUCTION AND ECONOMICS.

### THE FIFTEENTH INTERNATIONAL CONGRESS OF MEDICINE, LISBON, 1906.

A MEETING of the National Committee for Great Britain and Ireland of the Fifteenth International Medical Congress was held at the Medical Society's rooms on March 13, 1906. There were present Dr. Pavy, F.R.S., President, in the chair; Sir Thomas Barlow, Bart.; Sir Dyce Duckworth; Dr. Ferrier, F.R.S.; Colonel Sloggett, C.M.G.; Deputy Inspector-General Johnston, R.N.; Dr. Radcliffe Crocker; Mr. Jessop; Dr. Boyd Joll; Mr. Creasy; and the Honorary Secretaries, Dr. Clive Riviere and Mr. D'Arcy Power. The Hon. Secretaries reported that the railway companies had made important concessions to members attending the Congress. The British Association at Oporto invited twenty members of the Congress to dine in the Factory House on April 25, and they also extended hospitality to other members. The following communication was read from the German National Committee: The German National Committee beg to submit the following propositions to other National Committees, with the request that if they agree with them they should give expression to their views:—

PROPOSALS.

I.

An international Bureau should be established which should continue in activity in the intervals between the meetings. The Bureau should consist of the Presidencies of the previous and of the future Congresses and of the Presidencies of the several National Committees. The headquarters of the Bureau should be in Paris. Its business should be to maintain the continuity of the Congress, and to co-operate with the Organising Committee in the arrangement of the Programme as far as concerns the division into sections, and the choice of themes, of Reporters, Honorary Presidents, etc.

MOTIVES.

The need of a Court, which, by its international character, would be enabled to prevent or adjust differences of opinion between the Organising Committee and the Representatives of the various special branches of Medicine is seriously felt. At the same time the existence of such an international Court would make it possible to arrive at a general agreement as to the relation of the great International Congress with the International Special Congresses and the meetings of the Medical Societies in the different countries.

II.

In future the International Medical Congress should he held, not as hitherto, every three, but every five years.

MOTIVES.

It is now recognised on all sides that in recent years the International Medical Congress has diminished in importance. This is thought to be mainly due to the rapid sequence of these gatherings, and it is considered that the holding of meetings at longer intervals would not only make more careful preparation possible, but would encourage the devotion of greater energy and zeal to the solution of scientific problems by such authors who now have lost their interest for the meetings, owing to their too frequent recurrence. Moreover, less frequent meetings would make the choice of a place of meeting easier.

These propositions were passed after some discussion, but it was thought that the headquarters of the bureau should be in Brussels rather than in Paris. The British Committee was not prepared to make any suggestion as to the place of meeting of the sixteenth International Congress of Medicine. It was suggested that delegates going to the Congress should take academic costume. The proceedings ended with a hearty vote of thanks to the Medical Society for the use of their room.

